# The Vagaries of Fibromyomatous Tumors1Read before the Brant County Medical Association, Brantford, March, 1910.

**Published:** 1910-06

**Authors:** J. F. W. Ross

**Affiliations:** Toronto


### The Vagaries of Fibromyomatous Tumors[1]

BY J. F. W. ROSS, M.D., C.M., Toronto.
[*Canadian Practitioner and Review*, April, 1910.]

I REGRET that owing to the shortness of the notice it was impossible to prepare such an address as befits the occasion, and I offer my apologies for the imperfect presentation of the subject. I decided to look back into the years that have gone and endeavor to pick out some points that may prove of value in a consideration of the effects of fibromyomatous tumors upon the life history of women.

It was my privilege in 1878 to hold the position of House Surgeon at the Toronto General Hospital; that is now thirty-two years ago. It is interesting to watch the evolution of practice that has taken place in that time, and there is no department in surgery in which the changes have been more varied or the practice has been more improved than in the surgical treatment of fibromyomatous tumors.

During a pilgrimage to the Mecca of abdominal surgery, Birmingham, about the year 1889, I had the privilege of assisting that great pioneer, Mr. Lawson Tait, with many of his operations during a period of some months. While ovariotomy for the removal of ovarian tumors had been perfected so that the mortality was greatly reduced, the operation of hysterectomy for the removal of myomatous tumors was still in its infancy, and was accompanied by a high death-rate. This was so great that one Edinburgh surgeon looked about him for some other remedy than the knife, and began the use of the electric current, as advocated by Apostoli of Paris. After much investigation and careful trial this treatment was not satisfactory and proved to be at times dangerous, owing to the degenerative changes that

---

1. Read before the Brant County Medical Association, Brantford, March, 1910.

it was liable to set up in the tumors. Many of us looked about for some improvement of surgical technic, and eventually the operation at present adopted was evolved, and is now performed with as low a mortality, in skilled hands, as the operation of ovariotomy. It is my opinion that such operations should only be undertaken by men of special training, in well equipped operating rooms, under the most advantageous circumstances.

About the year 1890, the operation for removal of fibroid tumors of the fundus uteri was performed with the assistance of the Koeberle serre-noeud. Tait used as a primary precaution against hemorrhage a rope clamp, of which the rope was made to encircle the tissues about the cervix, after the peritoneum together with the bladder had been stripped down off the tumor surface; the Koeberle serre-noeud was placed so as to constrict the cervical structures, taking care to avoid the ureters, and after a severance of the ovarian and uterine arteries. The serre-noeud produced constriction of the stump and gangrene of its distal portion, notwithstanding the tanning effect produced by the application of perchloride of iron dissolved in glycerine. The patient did well until about the sixteenth or seventeenth day when a leakage took place from this foul mass into the general cavity of the peritoneum and a general septic peritonitis resulted, followed very shortly by the death of the patient. Even after recovery from such an operation an immense funnel-shaped granulating opening was left, through which a subsequent protrusion of the intestines took place; this was certainly anything but ideal surgery. We then became bolder and found that a direct dissection down on to the vessels enabled us to control the hemorrhage, and that the use of cat-gut sutures to the stump controled any little oozing that might be caused owing to a lack of ligation of the azygos vagina artery. The operation was then still further improved by a readjustment of the cut peritoneum over the surface of the stump, so that the stump became with the ligatures applied practically extraperitoneal. At first it was considered desirable to place a drainage tube in the culdesac of Douglas, but in later years even this was found to be unnecessary.

In my hands and those of my assistants these operations have now become entirely satisfactory and the mortality is almost nil. It is essential, of course, that the operator should see to it that all hemorrhage is properly controlled before the abdominal cavity is finally closed. There is another point in favor of operation,—namely, the fact that the tumors are not now allowed to grow to the gigantic proportions of those tumors formerly met with, and furthermore we do not have such extensive adhesions to deal with. As a consequence of the great success of the modern operations I fear the pendulum has swung rather too far to the other extreme, and that now young women are practically unsexed and are denied the opportunities of motherhood owing to the rather ruthless use of the knife on fibroid tumors as soon as they make their appearance. As fibroid tumors have vagarious ways it is desirable that we should be fully aware of these peculiar changes, in order that we may deal with these cases more intelligently. Let us take up the question systematically.

*Position.*—Fibroid tumors have been named according to their position. The classification adopted has been subperitoneal, intramural and submucous; we have also myomatous tumors growing from the myomatous structures about the cul-desac of Douglas in the broad ligament and in front towards the bladder; we have also fibroid tumors growing in either the anterior or posterior lip of the cervix.

(2) *Subperitoneal Tumors.*—Subperitoneal tumors seem to have certain characteristics not met with as frequently as in the others; they have a tendency to become pendunculated and may often be found roughened on the surface owing to calcareous degeneration, and as a consequence of this they may produce intraperitoneal dropsy that simulates the dropsy found accompanying malignant disease in the peritoneal cavity; they may become fixed to other organs and may eventually derive their blood supply through the adhesions in the new situation; they may become twisted and gangrenous or gangrenous owing to thrombosis of the vessels.

(b) *Intramural Tumors.*—Intramural tumors frequently give rise to menstrual pains and increased menstrual flow before they can be made out by the examining finger. When the uterus of a young unmarried woman is found somewhat enlarged and when this enlargement is accompanied by menstrual pain and increased flow, we must suspect the presence of an intramural fibroid. The ultimate destiny of the intramural variety is generally subperitoneal or submucous, as the constant contraction during menstruation, producing the pain already spoken of, tends to force the little nodule outwards or inwards.

(c) *Submucous Tumors.*—The submucous variety may be very small or may be large enough to simulate pregnancy at the fourth or fifth month, or even later. I have on two occasions been forced to dilate the cervix and introduce my finger into the uterus to satisfy my mind that the case was one of large submucous fibroid, filling the uterine cavity, before proceeding to amputate the uterus supra vaginally through the abdomen. I have seen similar cases in the practice of others, and on two occasions they simulated a pregnancy at full time. In each of these the abdomen was closed, as the operators felt they had made a mistake and that the cases were cases of pregnancy, and in each case a few days later the uterus was removed by a second operation, thus readily demonstrating how such submucous edematous growths can simulate pregnancy. Many of the submucous growths cause alarming hemorrhages and continued illhealth; eventually they may become polypoid and may be extruded from the uterine cavity into the vagina or forced outside the labia. I removed, at intervals covering several years, three such polypi from one patient.

(d) *Other Varieties.*—Those growing in the neighborhood of the culdesac of Douglas, either in front or behind the rectum, become a very serious bar to delivery, and I have performed cesarean section on three occasions, owing to the presence of this condition. Growths growing in the cervix, either in front or behind, may also become a serious menace to delivery; I have, however, seen such large growths gradually compressed and pushed above the pelvic brim and the patient delivered without mishap when we were quite prepared to perform cesarean section. Tumors growing in the anterior lip of the cervix produce serious bladder disturbances; retention of urine being one of the most common of these. The removal of growths situated either in front or behind the cervix or in the cervix itself is necessarily fraught with much danger; in front, damage to the ureters; behind, damage to the pelvic vessels. On one occasion I was forced to remove a tumor growing in the anterior cervical lip and causing retention of urine, and after the removal there was an opening in the vagina large enough to admit a fist. The patient was prepared for death upon the table, but fortunately rallied from the shock and made a good recovery, contrary to the expectations of all those connected with the case.

*Changes in the Tumor:*

Congestion.
Edema.
Cystic degeneration.
Necrosis with or without suppuration.
Calcareous change.
Malignant disease.
(a) Myxomatous degeneration.
(b) Sarcomatous degeneration.

*Congestion.*—No matter where situated in the pelvis, fibroid tumors are affected by the presence of an intra- or an extrauterine pregnancy and by menstruation; in either of these conditions the capsule of the tumor carrying the blood supply becomes much congested, and, as a consequence, for the time being, the tumors increase in size; owing to the fact that pregnancy is continued over a period of nine months the congestion remains continuous and the growth of the tumors is much greater; the menstrual congestion coming on but for a short time and ceasing does not add so rapidly to the size. In cases of pregnancy I have often considered that it is a race between the fetus and the growth as to which can grow the fastest. It is well to remember that ovarian tumors frequently cause a temporary cessation of menstruation, and that when such a temporary cessation of menstruation occurs in the presence of a fibroid tumor before the menopause, it is always due to pregnancy; this is an important point, as under such circumstances the uterine sound should not be used; it is oftentimes the unexpected that happens, and a woman with a fibroid tumor may go for years without becoming pregnant, and may then suddenly miss a menstrual period. When examination is made, the tumor will be found softened and considerably enlarged.

*Edema.*—The edema of fibromyomatous tumors is an extraordinary condition not seen anywhere else in the body; fluid is poured out in the meshes of the myomatous tissue and a separation of the long involuntary muscle fibers takes place; the tumor looks as if waterlogged, and on the surface has a sense of false fluctuation; this sense of fluctuation so closely simulates genuine fluctuation that the presence of disseminated and not encysted fluid can oftentimes only be made out by an incision into the tumor. The cause of this edema outside of that form that accompanies myxomatous degeneration is not very well understood, unless it is due to an obstruction of the blood supply or a damming back of the venous circulation. I have seen one such tumor 60 pounds in weight; I saw another enormous tumor removed from a woman in England, where it seemed as if the woman was peeled away from the tumor, and I have myself removed a tumor of upwards of 40 pounds in weight. We do not see these edematous tumors as frequently now as we did a few years ago, owing to the fact, as already stated, that hysterectomy and ablation of the growth is not fraught with such a high mortality; the mortality having now been reduced, in skilled hands, to equal that of ovariotomy. It is extremely difficult to say when the edematous tumors are myxomatous and malignant and when they are simply myxomatous and innocent. I always feel suspicious of the malignancy of an edematous fibromyoma. In the cases in which I have seen the disease return in the form of pseudo myxoma, in the peritoneum there is nothing to indicate that the tumor was malignant at the time of its removal. A microscopical examination should be made of all edematous fibromyomata removed. Enormous edematous fibromyomatous tumors may entirely disappear, or almost entirely, subsequent to the onset of the menopause. I have a distinct recollection of two patients who had such edematous tumors. One of them declined to submit to surgical measures until after the marriage of her daughter. She was confined to the house for about two years with terribly swollen limbs and enormously distended abdomen; she subsequently recovered perfect health, and it is not long since I met her, a very active woman for her age. The other woman was an invalid for several years, and the tumor in her case similarly disappeared and she was restored to health. Of course it is a terrible penalty to pay and we do not always have such a favorable termination, but these patients were seen before the days when supra vaginal amputation of the uterus had such a small mortality as at the present time, and surgeon and patient alike dreaded operative interference.

*Cystic Degeneration.*—The cystic degeneration of these tumors is not a true cystic degeneration originating in glandular structure. Small hemorrhages take place here and there into the substance of the tumor and these hemorrhages are followed by the formation of cysts. There seems to be a difference between ordinary cystic degeneration of fibromyomatous tumors and the true fibrocystic tumors of the uterus. Cystic tumors of the uterus are very rarely met with, whereas cystic degeneration of fibroid tumors is not infrequent; in either case these growths require to be removed, as they have a tendency to increase in size or to undergo necrotic change. I have seen but two cases of marked fibrocystic tumors of the uterus, and we have not a single specimen in our pathological museum. I removed one such tumor from a negress in one of the hospitals in Pittsburg some years ago, and the other tumor I saw removed by Mr. Lawson Tait.

*Necrosis With or Without Suppuration.*—This is a very serious condition and imperils life. The first case of necrosis of a fibroid tumor I met with was one into which a hand and arm had to be introduced through the vagina and up into the tumor to dislodge the broken down tissue. The patient made a very slow but excellent recovery. Necrosis occurs as a consequence of thrombosis of the bloodvessels; this thrombosis seems to be produced by excessive congestion and increased coagulation of the blood, such as occurs in pregnancy; by pressure produced by uterine contractions subsequent to the administration of ergot; by constriction of the pedicle of a myomatous polypus, and by a disturbance of the parts such as is unavoidable in the performance of an abdominal operation. I have seen numbers of cases of gangrene and necrosis of fibromyomatous tumors before and after delivery; when it occurs before the delivery of the patient the condition is particularly dangerous; these patients are liable to become pyemic and to lose their lives. If delivery has taken place there is then a chance that the contents of the sloughing tumor may be extruded and that the sloughing tumor may be reached by the surgeon. The treatment under such conditions should consist in as thorough a cleansing of the parts as possible, and a removal of as much of the necrotic tissue as possible. The antiseptic that is perhaps most serviceable is bichloride of mercury; this should be used as a douche into the uterine cavity once or twice a day, and perhaps it may be considered advisable to pack the uterine cavity with iodoform gauze; a hysterectomy under such circumstances is not to be thought of; to open up by incision a large area necessary in performing this operation in the presence of a fetid and extremely poisonous gangrene is very unwise. When, during pregnancy, a tumor becomes necrotic, an abdominal hysterectomy will give the best results, and is then indicated. Operation must not be long delayed if we hope to save the patient; necrosis in such cases is generally indicated by a sudden tenderness over the tumor, accompanied in all probability with chills, together with increased pulse rate and sudden rapid increase in the size of the growth.

The necrosis of fibromyomatous tumors is liable to occur after the removal of ovaries and tubes, or, in other words, after the operation of oöphorectomy for fibroid; sometimes the tumor becomes inflamed under such circumstances, but does not become completely necrosed. After the removal of a fibroid tumor I have been surprised on a number of occasions to find evidence of old necrotic changes. When polypi are extruded from the cervix or from the vagina they are liable to become gangrenous. In the early stages of such gangrene, the tumor simulates very closely malignant growth, and it is necessary for the surgeon to discriminate between the two; in either case there is a very considerable malodorous discharge, frequently tinged with blood poured out from ulcerated areas. When such tumors have been removed the differential diagnosis can readily be made by making an incision into the tumor substance, and by examining a section of the tumor under the microscope. These polypi can be very readily removed, as the thrombosis of the vessels at the pedicle prevents hemorrhage, provided the pedicle is separated below the upper limit of the occlusion of the vessels. I have seen such black tumors as large as a man's head, between the thighs, entirely outside the labia. The so-called red degeneration is nothing more nor less than the early stage of necrotic change; the tissue has the appearance of being acutely inflamed and hence looks red.

*Calcareous Degeneration.*—Calcareous degeneration is more frequently found in the subperitoneal variety of fibromyomatous growths; these growths become roughened on the surface, and owing to the presence of intraperitoneal fluid they are liable to simulate malignant disease; they may be found bobbing about in the fluid, and may as a consequence feel much like fetal parts. I have several times operated on such growths, when a diagnosis of probable malignancy had been made, and we were afraid that operative interference would be useless; under such circumstances it is always wiser to open the abdomen. When the tumors are removed, often by means of ligatures round the pedicle, the peritoneal dropsy disappears and the patients resume a normal condition.

*Malignant Change.*—Myxomatous degeneration in fibromyomatous tumors is in my experience fairly common in proportion to the number of cases that undergo malignant change. I have never seen any other malignant change except myxomatous degeneration and sarcomatous degeneration; myxomatous degeneration is particularly prone to recur after removal of the tumor; this recurrence presents some interesting features; the peritoneal surface of the intestines and the parietal walls appear as if injected with gelatine, the bowels become stiffened and partly rigid as a consequence of this thickening of the coats; the disease has been called pseudo-myxoma-peritonei. The patients gradually become weaker and weaker and finally die with some of the symptoms of intestinal obstruction. When sarcomatous degeneration occurs in the tumor the tumor becomes rapidly enlarged, there may be some elevation of temperature, the patient's general health is not particularly affected, and there are no other changes to be noted; it is only after the tumor has been removed and has been cut into that the sarcomatous change is determined; the microscope then completes the diagnosis. After removal of the tumor the patients may be free from recurrence for a considerable time, or the disease may recur at an early date. I have never seen carcinomatous degeneration of a fibroid tumor, but feel satisfied that when carcinomatous disease is met with in the presence of a fibroid tumor it is merely a coincidence and has nothing to do with the presence of the fibroid. I have always found the carcinomatous growth growing definitely from the glandular structures of the endometrium.

In the presence of pregnancy fibromyomatous tumors do not seem to have any particular tendency to produce miscarriage.

When it is considered desirable to empty the uterus, owing to existing circumstances, it is usually necessary for the surgeon to procure an abortion. I have found it desirable after one or more consultations to produce miscarriage in a number of cases. If a young woman has had no children and is troubled with a small myomatous tumor, I believe that in most cases when the tumor has reached an important size that miscarriage should be produced, and, as a consequence, she is given the benefit of the subsequent involution. Many fibromyomatous tumors disappear after the first miscarriage; I have seen them disappear after labor at full term: in fact they disappear, or almost disappear, as a consequence of the process of involution. If a woman has not had progeny and on the other hand is willing and anxious to submit to cesarean or Porro cesarean operation at any time when it is found to be necessary or desirable, in order that the life of the mother or of the mother and child shall be saved, her wish should be gratified. Under modern conditions cesarean operation may be safely performed, but it must be remembered that it may be necessary, in the presence of fibroid tumors, to perform the Porro cesarean operation in order to control hemorrhage and thus remove from the woman all chance of subsequent motherhood. I have advised young women with fibroid tumors of small size to become married as a prophylactic measure, with the hope that either childbirth or miscarriage would be beneficial by checking the growth of the tumor. To illustrate my point, let me state further that my first experience was obtained by a rather rude awakening. A missionary lady from Africa, between 35 and 40 years of age, married. I saw her, in consultation with the late Dr. J. E. Graham, and we found a pregnancy nestled in between three large fibroid tumors; miscarriage was produced, and I asked her to return at a subsequent date, in order that I might remove the uterus. During the process of involution she was advised to take a certain treatment, and the treatment got the credit for what occurred; the tumors almost entirely disappeared, she again became pregnant and was delivered of a living child; surely this should be a warning to those who advise the removal of small myomatous growths. It is argued that these growths should be removed, for fear that they may become malignant; I consider that this is erroneous teaching, as the growths seldom become malignant, and to prevent carcinomatous disease of the uterus we would be compelled to remove the organ from every woman. After the childbearing period is passed and after the growth has reached such proportions that the chances of motherhood are nil, then I believe the surgeon is justified in operating. In patients who have been suffering great loss of blood from time to time I have been able to tide them over until the coming polypus has made its appearance, when its removal cures the patient, relieves her of her symptoms, and restores her to health.

And now, in conclusion, let me say that as motherhood is the rounding out of the life history of woman, and as the uterus is an organ that is essential to this end, we must, as physicians and surgeons, see to it that it is not ruthlessly mutilated by the knife, but rather that every effort shall be put forth to preserve it intact, and to improve the health and save the life of the patient.

481 Sherbourne Street.

